# Cohort study on living arrangements of older men and women and risk for basic activities of daily living disability: findings from the AGES project

**DOI:** 10.1186/s12877-017-0580-7

**Published:** 2017-08-16

**Authors:** Tami Saito, Chiyoe Murata, Jun Aida, Katsunori Kondo

**Affiliations:** 1 Department of Social Science, National Center for Geriatrics and Gerontology, 7-430 Morioka-cho, Obu, Aichi 474-8511 Japan; 2 Department of International and Community Oral Health, Tohoku University Graduate School of Dentistry, Seiryo-machi, Aoba-ku, Sendai, Miyagi 980-8575 Japan; 3 Center for Preventive Medical Science, Chiba University, 1-8-1 Inohana, Chuo-ku, Chiba, 260-8670 Japan; 4 Department of Gerontological Evaluation, National Center for Geriatrics and Gerontology, 7-430 Morioka-cho, Obu,, Aichi 474-8511 Japan

**Keywords:** Basic activities of daily living, Living arrangements, Longitudinal study, Japanese

## Abstract

**Background:**

Living arrangements of older adults have changed worldwide with increasing solitary and non-spouse households, which could affect social care systems. However, the relationship between these households and disability onset has remained unclear. We examined the relationship between living arrangements and the onset of basic activities of daily living disability in older adults, with a focus on gender differences and cohabitation status of those without a spouse.

**Methods:**

Data from 6600 men and 6868 women aged 65 years or older without disability were obtained from the Aichi Gerontological Evaluation Study Project in Japan. Onset of disability was followed for 9.4 years. Disability was assessed based on Long-term Care Insurance System registration. A hierarchical Cox proportional hazards model was conducted to examine the risk of living alone and living only with non-spousal cohabitants compared to those living with spouses.

**Results:**

Men living only with non-spousal cohabitants and those living alone were significantly more likely to develop disability after controlling for health and other covariates (hazard ratio = 1.38 and 1.45, respectively), while a significant difference was found only for women living alone (hazard ratio = 1.19). The risk of living with non-spousal cohabitants was marginally stronger in men, indicated by the interaction effect model (*p* = .08). A series of hierarchical analyses showed that social support exchange explained 24.4% and 15.8% of the excess risk of disability onset in men living alone and those living only with non-spousal cohabitants, respectively. A subsequent analysis also showed that support provision by older adults more greatly explained such excess risk than receiving support from others.

**Conclusions:**

Older men without spouses were more likely to develop disability onset regardless of cohabitants. Health professionals should consider programs that enhance social support exchange, particularly support provision by older adults who are at risk of disability.

**Electronic supplementary material:**

The online version of this article (doi:10.1186/s12877-017-0580-7) contains supplementary material, which is available to authorized users.

## Background

Prevention of functional disability among older individuals and related costs to the healthcare system are a pressing issue in aging societies [1, 2]. In countries with the most progressed aging populations, such as Japan, the proportion of older people is expected to reach 40% in 2050 [3].

Living arrangements of older adults have changed dramatically over time [4]. The proportion of older adults living alone is increasing worldwide [5], which could affect care provision by the community [6] and necessitates more detailed focus on their health problems and needs. In addition, the proportion of older adults without spouses living in the household could increase in the future. For instance, in the United States, about one third of baby boomers were unmarried [7]. Such changes in living arrangements imply the growing importance of examining the role of non-spousal networks in older adults’ health.

Many longitudinal studies have reported marital advantage regarding mortality and morbidity [8–12]; in contrast, relatively few studies have examined the relationship between functional disability and living with non-spousal cohabitants [11–17]. The presence of non-spousal family members of the unmarried could compensate for the lack of marital protection for older adults [18], while solitary living could promote independence among older adults [19]. Previous longitudinal studies have shown that older adults who lived only with non-spousal family members were more likely to develop functional decline compared to those who lived with spouses [12, 14, 15] or those who lived alone [11, 13, 16, 17]. Regarding those living alone, there have been inconsistent findings of comparable risk [11, 15, 16], excess risk [20], or less risk [14] compared to older adults living with a spouse.

Based on these findings, several points are worth further consideration. First, gender differences could exist in the association between living arrangements and onset of disability. Studies have shown more benefits for men living with a spouse compared to women for mortality [8, 21] and morbidity [10]. The association between living with non-spousal cohabitants and health could also differ by gender, since intimate relationships were more likely to be limited to a wife in older men, while older women relied on more diverse individuals such as their children [22]. However, few longitudinal studies have examined gender differences in the relationship between living arrangements and disability [11, 20].

Second, several factors could mediate the association between living arrangements and onset of disability. In studies on marital status and health, “marital protection [8],” referring to benefits obtained because of marriage, such as economic resources, social resources such as social support networks [7, 8, 10], and control over health-related behaviors [10], affects health in older adults [8, 13]. This has been observed even when considering the association of “marital selection,” a precursor to marital status [8]. Also, mental disorders in middle-aged adults differed by types of living arrangement, partially due to differential social support and unhealthy behaviors [10]. However, it remains unclear which factors mediate the association between living arrangements and onset of disability.

Third, needs-driven cohabitation among older adults, meaning those needing support tend to live with family members, particularly with their adult children [23], could confound the findings [17]. In Japan, living arrangements of older adults are relatively stable [24] because of low rates of needs-driven cohabitation [25] and remarriage [26]. Takagi and colleagues examined types of cohabitation with children and indicated that among older adults residing with their children, nearly 80% resided with their children their entire life [25]. This suggests that most older adults who reside with their children started living with them before the onset of health decline. Thus, reverse causation is less likely to be an issue as compared to Western countries, where needs-driven cohabitation is dominant [23].

The aim of this study was to examine the relationship between living arrangements and the onset of BADL disability among older adults with a focus on gender differences and cohabitation status in those living without a spouse. Following this, we also examined how social support and health-related behaviors mediated these associations.

## Methods

### Participants

Data were obtained from the Aichi Gerontological Evaluation Study (AGES) cohort dataset, which was part of the Japan Gerontological Evaluation Study (JAGES) project. A self-administered questionnaire survey was conducted in October 2003 with 33,152 people aged 65 years or older who were not eligible to utilize Long-term Care Insurance (LTCI) system services and who were selected through simple randomization (6 municipalities) or complete enumeration (4 small-scale municipalities) in Aichi Prefecture, Central Japan (response rate = 52.1%). The details of the survey were shown elsewhere [1, 27].

Survey data from 15,313 respondents who provided information for identification by the LTCI system were linked to the LTCI records dataset for a follow-up period of 3436 days (9.4 years) from November 1, 2003. We excluded data from 401 respondents who had qualified for LTCI benefits with a level 2 or higher rating by October 31, 2004, 332 who did not apply for LTCI benefits despite having BADL limitations, and 806 who did not provide information on the BADL items, to avoid the problem of reverse causation. We also excluded data for 306 cases for which information on living arrangements was not provided. Data for a total of 13,468 respondents (6600 men and 6868 women) were finally included in the analysis.

### Measurements

#### Outcome

We collected information on the onset of BADL decline from the LTCI records administrated by municipalities. The LTCI system classifies frail older adults into seven levels (Support need levels 1 and 2 and Care need levels 1 to 5; a larger number indicates a more severe level) using a nationally standardized and validated algorithm. The levels are solely determined according to older adults’ physical and mental care needs, regardless of informal care provided to the recipients [28] and assessed by both computer-based and home-visit interviews by a trained healthcare professional and examination by a primary physician [29]. In the computer-based assessment, time required for care is calculated according to nine categories of care needs such as five domains of BADL care (bathing, eating, toileting, dressing, and transferring), assistance with instrumental activities of daily living (IADL), behavioral problems, rehabilitation, and medical services [28]. In our study, BADL decline was defined as a new registration within the LTCI records with a care-needs level of 2 or above, which requires 50 min of care or longer per day and nearly corresponds to the need for any type of BADL care [29]. More detailed information was shown in another study [28].

#### Living Arrangements

Japanese families have been traditionally based on the “stem family” system, indicating cohabitation of parent(s) and one of their children (typically, the eldest son and his wife) [30]. Although a recent trend shows an increase in solitary households, the majority of older adults live with their spouse and/or children [31]. Since our focus was on examining the risk of cohabitation status of older adults without spouses, we created a living arrangement variable with three categories: living with a spouse, living without a spouse but with at least one non-spousal cohabitant, and living alone. Previous studies in Asian countries [11, 15] showed no significant differences based on the presence of children among those who lived with a spouse, suggesting that living with a spouse could comprise one category regardless of the presence of other family members. Regarding older adults living only with non-spousal cohabitants, about 90% lived with their children, grandchildren, or other blood relatives in this study. Although it is possible that older adults living only with in-law families differ psychosocially from those living with blood relatives, our preparatory analysis showed almost no significant differences between these groups, except for education and income. Therefore, we regarded them as a group. Additionally, a total of 20 cases among 13,468 respondents lived only with non-relatives. Those people were included in the category of older adults living only with non-spousal cohabitants.

#### Mediators

We regarded health-related behaviors and social support as possible mediators in the relationship between living arrangements and onset of BADL decline according to the context of marital protection [7, 8, 10]. In terms of health-related behaviors, we assessed smoking habits (none vs. past/current), alcohol consumption (none vs. yes), and body mass index, which was calculated using the respondent’s self-rated height and weight, and categorized into “less than 18.5 kg/m^2^,” “18.5–24.9 kg/m^2^,” and “25.0 kg/m^2^ or over.” In addition, daily walking time (less than 30 min vs. 30 min or longer) was assessed as a potential physical activity mediator, since walking was one of the most popular physical activities in Japanese older adults [32] and known as a predictor of physical function [33]. Regarding social support, we assessed emotional support received (“Do you have someone to listen to your concerns or complaints?”), emotional support provided (“Do you listen to someone’s concerns and complaints?”), instrumental support received (“Do you have someone who looks after you when you are sick and confined to a bed for a few days?”), and instrumental support provided (“Do you look after someone when he/she is sick and confined to a bed for a few days?”). Each support variable had “yes” or “no” response options.

#### Covariates

Covariates in this study were selected according to previous studies on risk factors for functional decline [34]: age, education, income, and baseline health status as potential precursors of living arrangements of older adults [23]. In terms of health variables, we assessed self-rated health, presence of illness, depression, IADL, and subjective cognitive complaints (SCC). Self-rated health was assessed using one question: “How do you rate your health?” Response options ranged from excellent to poor, and were dichotomized into two categories (excellent/good vs. fair/poor). Presence of illness was assessed in terms of whether participants had at least one illness such as cancer, heart disease, stroke, hypertension, diabetes, obesity, hyperlipidemia, osteoporosis, arthritis, trauma, respiratory illness, gastrointestinal illness, liver illness, mental illness, dysphagia, visual/hearing impairment, or incontinence. Depression was assessed with a 15-item Japanese version of the Geriatric Depression Scale [35]. Scores on the scale were categorized into three groups: “no depression (0–4 points),” “depressive tendency (5–9),” and “depression (10 and above).” We assessed IADL using a five-item subscale from the Tokyo Metropolitan Institute of Gerontology Higher Competence Scale. The scale was developed based on Lawton’s model of competence [36] and its validity and reliability were confirmed [37]. We dichotomized respondents based on their having difficulty with at least one item, for instance, shopping for daily necessities. We assessed SCC using one item asking respondents if they often perceived themselves to be disoriented. Equivalized household income (low, middle, or high), years of education (less than 10 years, or 10 years or more), age in years, and gender (male or female) were also assessed. All mediators and covariates included a missing category, except for age and gender.

### Analysis

All analyses were conducted by gender because we hypothesized that the relationships between living arrangements and BADL disability would differ by gender. After presenting descriptive statistics and differences for each variable among the three living arrangement categories, we examined the relationship between living arrangements and the onset of BADL disability using a Cox proportional hazards model by entering the group of covariates and mediators hierarchically. First, only age was controlled (Model 1). Second, household income, education, and health variables were added to Model 1 (Model 2) to examine the relationship of living arrangements, excluding differences based on the precursors. Additionally, we conducted a series of sub-analyses that examined the differences between the two non-spouse household groups in men and women. Further, to examine gender differences in the relationship between living arrangements and onset of BADL disability, we entered the cross-product terms of living with cohabitants or living alone by gender using the whole sample.

The next three models were employed to examine the influence of mediators on the relationship between living arrangements and BADL disability onset. We added each group of health-related behavior variables and social support variables to Model 2 (Models 3 and 4). Finally, all covariates were entered with living arrangements in the analytical model (Model 5). As a sensitivity analysis, we conducted mediation analysis for Models 3 and 4 to estimate mediation effects of health-related behavior variables (Model 3) and social support variables (Model 4). For this analysis, we applied logistic regression models instead of Cox proportional hazards models due to the limitation of the software program. Stata command “ldecomp” was used for the mediation analysis [38].

Although missing cases for each covariate were modest (24.8% at the most), 41.1% of the analyzed respondent data had a missing score for at least one covariate. Therefore, in those multivariate models, we performed a multiple imputation by chained equations under the assumption of missing at random. We generated 20 datasets, analyzed them separately, and pooled the estimates and standardized errors applying Rubin’s rules [39]. For mediation analysis, complete case analysis was applied. We regarded respondents who died or who were lost to follow-up due to relocation before the onset of BADL disability as censored cases. All analyses were conducted using IBM SPSS 22.0 J for Windows (IBM Japan Ltd., Tokyo, Japan) and STATA SE version 14.1 (Stata Corp., College Station, TX, USA). Statistical significance was set at *p* < .05.

## Results

Tables 1 and 2 show the respondents’ characteristics by living arrangement status for men and women. For men, 85.5% lived with a spouse, 10.3% lived only with non-spousal cohabitants, and 4.2% lived alone; fewer women lived with a spouse (54.1%). Those living only with non-spousal cohabitants were older than those living with spouses for both men and women. Men living alone were more likely to have a depressive tendency or depression than those living only with non-spousal cohabitants or those living with a spouse. In contrast, IADL difficulty was less likely among men living alone compared to those living with non-spousal cohabitants or those living with a spouse. Men living alone were less likely to receive and provide emotional and instrumental support than those living with a spouse. For women, those living alone were less likely to receive or provide instrumental support than those with a spouse.

**Table 1:** Respondent characteristics by living arrangements for men (*N* = 6600)

Variables and categories	Total	With spouse	With non-spousal cohabitants	Living alone	
	n (%)	n (%)	n (%)	n (%)	*p* ^a^
Living arrangements					
Living with spouse	5645 (85.5)	―	―	―	―
Living with non-spousal cohabitants	677 (10.3)	―	―	―	―
Living alone	278 (4.2)	―	―	―	―
Age					
65–69	2567 (38.9)	2308 (40.9)	173 (25.6)	86 (30.9)	*p* < .001
70–74	2044 (31.0)	1767 (31.3)	191 (28.2)	86 (30.9)	
75–79	1275 (19.3)	1048 (18.6)	159 (23.5)	68 (24.5)	
80–84	526 (8.0)	409 (7.2)	94 (13.9)	23 (8.3)	
85 and older	188 (2.8)	113 (2.0)	60 (8.9)	15 (5.4)	
Years of education					
< 10	3603 (54.6)	2991 (53.0)	458 (67.7)	154 (55.4)	*p* < .001
≥ 10	2950 (44.7)	2618 (46.4)	211 (31.2)	121 (43.5)	
Missing	47 (0.7)	36 (0.6)	8 (1.2)	3 (1.1)	
Household income					
Low	2263 (34.3)	1981 (35.1)	204 (30.1)	78 (28.1)	*p* < .001
Middle	3022 (45.8)	2633 (46.6)	267 (39.4)	122 (43.9)	
High	731 (11.1)	616 (10.9)	95 (14.0)	20 (7.2)	
Missing	54 (8.8)	415 (7.4)	111 (16.4)	58 (20.9)	
Self-rated health					
Excellent/good	4879 (73.9)	4229 (74.9)	453 (66.9)	197 (70.9)	*p* < .001
Fair/poor	1659 (25.1)	1370 (24.3)	211 (31.2)	78 (28.1)	
Missing	62 (0.9)	46 (0.8)	13 (1.9)	3 (1.1)	
Presence of illness					
No	1204 (18.2)	1036 (18.4)	115 (17.0)	53 (19.1)	*p* = .239
Yes	5181 (78.5)	4436 (78.6)	531 (78.4)	214 (77.0)	
Missing	215 (3.3)	173 (3.1)	31 (4.6)	11 (4.0)	
Geriatric Depression Scale					
No depression	4304 (65.2)	3784 (67.0)	398 (58.8)	122 (43.9)	*p* < .001
Depressive tendency	1264 (19.2)	1037 (18.4)	153 (22.6)	74 (26.6)	
Depression	364 (5.5)	273 (4.8)	51 (7.5)	40 (14.4)	
Missing	668 (10.1)	551 (9.8)	75 (11.1)	42 (15.1)	
Instrumental activities of daily living					
Without difficulty	4935(74.8)	4212 (74.7)	483 (71.3)	238 (85.6)	*p* < .001
With difficulty	1455 (22.0)	1256 (22.2)	165 (23.8)	34 (12.2)	
Missing	210 (3.2)	175 (3.0)	29 (4.3)	6 (2.2)	
Subjective cognitive complaints					
No	5597 (84.8)	4811 (85.2)	549 (81.1)	237 (85.3)	*p* = .023
Yes	891 (13.5)	737 (13.1)	114 (16.8)	40 (14.4)	
Missing	112 (1.7)	97 (1.7)	14 (2.1)	1 (0.4)	
Body mass index					
< 18.5	435 (6.6)	337 (6.0)	71 (10.5)	27 (9.7)	*p* < .001
18.5–24.9	4685 (71.0)	4036 (71.5)	454 (67.1)	195 (70.1)	
≥ 25	1320 (20.0)	1147 (20.3)	125 (18.5)	48 (17.3)	
Missing	160 (2.4)	125 (2.2)	27 (4.0)	8 (2.9)	
Alcohol consumption					
No	2700 (40.9)	2258 (40.0)	317 (46.8)	125 (45.0)	*p* = .007
Yes	3812 (57.8)	3312 (58.7)	350 (51.7)	150 (54.0)	
Missing	88 (1.3)	75 (1.3)	10 (1.5)	3 (1.1)	
Smoking habit					
None	1737 (26.3)	1494 (26.5)	174 (25.7)	69 (24.8)	*p* = .073
Past/current	4657 (70.6)	3989 (70.7)	473 (69.9)	195 (70.1)	
Missing	206 (3.1)	162 (2.9)	30 (4.4)	14 (5.0)	
Daily walking time					
≥ 30 min	4105 (62.2)	3556 (630)	383 (56.6)	166 (59.7)	*p* = .015
< 30 min	2069 (31.3)	1735 (30.7)	239 (35.3)	95 (34.2)	
Missing	426 (6.5)	354 (6.3)	55 (8.1)	17 (6.1)	
Emotional support received					
Yes	5471 (82.9)	4776 (84.6)	505 (74.6)	190 (68.3)	*p* < .001
No	858 (13.0)	657 (11.6)	126 (18.6)	75 (27.0)	
Missing	271 (4.1)	212 (3.8)	46 (6.8)	13 (4.7)	
Emotional support provided					
Yes	5179 (78.5)	4532 (80.3)	469 (69.3)	178 (64.0)	*p* < .001
No	1113 (16.9)	866 (15.3)	163 (24.1)	84 (30.2)	
Missing	308 (4.7)	247 (4.4)	45 (6.6)	16 (5.8)	
Instrumental support received					
Yes	6157 (933)	5369 (95.1)	610 (90.1)	178 (64.0)	*p* < .001
No	239 (3.6)	110 (1.9)	41 (6.1)	88 (31.7)	
Missing	204 (3.1)	166 (2.9)	26 (3.8)	12 (4.3)	
Instrumental support provided					
Yes	5904 (89.5)	5221 (92.5)	518 (76.5)	165 (59.4)	*p* < .001
No	435 (6.6)	223 (4.0)	116 (17.1)	96 (34.5)	
Missing	261 (4.0)	201 (3.6)	43 (6.4)	17 (6.1)	

^a^A chi-square test was used to examine differences among living arrangement categories

**Table 2:** Respondent characteristics by living arrangements for women (*N* = 6868)

Variables and categories	Total	With spouse	With non-spousal cohabitants	Living alone	
	n (%)	n (%)	n (%)	n (%)	*p* ^a^
Living arrangements					
Living with spouse	3716 (54.1)	―	―	―	―
Living with non-spousal cohabitants	2076 (30.2)	―	―	―	―
Living alone	1076 (15.7)	―	―	―	―
Age					
65–69	2417 (35.2)	1724 (46.4)	426 (20.5)	267 (24.8)	*p* < .001
70–74	1993 (29.0)	1171 (31.5)	480 (23.1)	342 (31.8)	
75–79	1433 (20.9)	632 (17.0)	536 (25.8)	265 (24.6)	
80–84	701 (10.2)	166 (4.5)	388 (18.7)	147 (13.7)	
85 and older	324 (4.7)	23 (0.6)	246 (11.8)	55 (5.1)	
Years of education					
< 10	4294 (62.5)	2227 (59.9)	1430 (68.9)	637 (59.2)	*p* < .001
≥ 10	2495 (36.3)	1460 (39.3)	614 (29.6)	421 (39.1)	
Missing	79 (1.2)	29 (0.8)	32 (1.5)	18 (1.7)	
Household income					
Low	2257 (32.9)	1276 (34.3)	539 (26.0)	442 (41.1)	*p* < .001
Middle	2269 (33.0)	1498 (40.3)	545 (26.3)	226 (21.0)	
High	637 (9.3)	330 (8.9)	279 (13.4)	28 (2.6)	
Missing	1705 (24.8)	612 (16.5)	713 (34.3)	380 (35.3)	
Self-rated health					
Excellent/good	4994 (72.7)	2721 (73.2)	1493 (71.9)	780 (72.5)	*p* = .118
Fair/poor	1752 (25.5)	941 (25.3)	533 (25.7)	278 (25.8)	
Missing	122 (1.8)	54 (1.5)	50 (2.4)	18 (1.7)	
Presence of illness					
No	1124 (16.4)	649 (17.5)	326 (15.7)	149 (13.8)	*p* = .013
Yes	5393 (78.5)	2896 (77.9)	1628 (78.4)	869 (80.8)	
Missing	351 (5.1)	171 (4.6)	122 (5.9)	58 (5.4)	
Geriatric Depression Scale					
No depression	3961 (57.7)	2243 (60.4)	1180 (56.8)	538 (50.0)	*p* < .001
Depressive tendency	1319 (19.2)	699 (18.8)	376 (18.1)	244 (22.7)	
Depression	384 (5.6)	187 (5.0)	128 (6.2)	69 (6.4)	
Missing	1204 (17.5)	587 (15.8)	392 (18.9)	225 (20.9)	
Instrumental activities of daily living					
Without difficulty	5630 (82.0)	3229 (86.9)	1474 (71.0)	927 (86.2)	*p* < .001
With difficulty	1010 (14.7)	386 (10.3)	506 (24.4)	118 (11.0)	
Missing	228 (3.3)	101 (2.5)	96 (4.6)	31 (2.9)	
Subjective cognitive complaints					
No	5673 (82.6)	3179 (85.5)	1601 (77.1)	893 (83.0)	*p* < .001
Yes	1035 (15.1)	464 (12.5)	414 (19.9)	157 (14.6)	
Missing	160 (2.3)	73 (1.9)	61 (2.9)	26 (2.4)	
Body mass index					
< 18.5	531 (7.7)	245 (6.6)	190 (9.2)	96 (8.9)	*p* < .001
18.5–24.9	4497 (65.5)	2498 (67.2)	1289 (62.1)	710 (66.0)	
≥ 25	1523 (22.2)	860 (23.1)	452 (21.8)	211 (19.6)	
Missing	317 (4.6)	113 (3.0)	145 (7.0)	59 (5.5)	
Alcohol consumption					
No	5882 (85.6)	3157 (85.0)	1818 (87.6)	907 (84.3)	*p* < .001
Yes	848 (12.3)	508 (13.7)	202 (9.7)	138 (12.8)	
Missing	138 (2.0)	51 (1.4)	56 (2.7)	31 (2.9)	
Smoking habit					
None	6176 (89.9)	3387 (91.13)	1845 (88.9)	944 (87.7)	*p* < .001
Past/current	411 (6.0)	206 (5.5)	122 (5.9)	83 (7.7)	
Missing	281 (4.1)	123 (3.3)	109 (5.3)	49 (4.6)	
Daily walking time					
≥ 30 min	3768 (54.9)	2078 (55.9)	1133 (54.6)	557 (51.8)	*p* = .015
< 30 min	2187 (31.8)	1147 (30.9)	651 (31.4)	389 (36.2)	
Missing	913 (13.3)	491 (13.2)	292 (14.1)	130 (12.1)	
Emotional support received					
Yes	6189 (90.1)	3402 (91.6)	1856 (89.4)	931 (86.5)	*p* < .001
No	402 (5.9)	183 (4.9)	119 (5.7)	100 (9.3)	
Missing	277 (4.0)	131 (3.5)	101 (4.9)	45 (4.2)	
Emotional support provided					
Yes	5587 (81.3)	3155 (84.9)	1566 (75.4)	866 (80.5)	*p* < .001
No	887 (12.9)	385 (10.4)	363 (17.5)	139 (12.9)	
Missing	394 (5.7)	176 (4.7)	147 (7.1)	71 (6.6)	
Instrumental support received					
Yes	6119 (89.1)	3415 (91.9)	1868 (90.0)	836 (77.7)	*p* < .001
No	502 (7.3)	180 (4.8)	125 (6.0)	197 (18.3)	
Missing	247 (3.6)	121 (3.3)	83 (4.0)	43 (4.0)	
Instrumental support provided					
Yes	5996 (87.3)	3440 (92.6)	1732 (83.4)	824 (76.6)	*p* < .001
No	496 (7.2)	120 (3.2)	205 (9.9)	171 (15.9)	
Missing	376 (5.5)	156 (4.2)	139 (6.7)	81 (7.5)	

^a^A chi-square test was used to examine differences among living arrangement categories

Table 3 shows the incidence rate of the onset of BADL disability during the 9.4-year follow-up period in each category of the explanatory variables. A total of 1108 men and 1248 women showed new onset of disability, with incidence rates per 1000 person-years being 21.4 and 22.3, respectively, for men and women. In men, the incidence rates were 19.4, 35.6, and 32.4, in those with a spouse, living only with non-spousal cohabitants, and living alone, respectively. In women, the rates were 15.0, 34.0, and 27.8, in those who lived with a spouse, those without a spouse and with non-spousal cohabitants, and those living alone, respectively. The incidence rate of onset of BADL disability in those aged 85 and older was more than 10 times higher than those aged 65 to 69 in both men and women.

**Table 3:** Incidence rate of basic activities of daily living disability onset during the follow-up period

Variables	Categories	Men			Women		
		Incidence/person-year	Incidence rate per 1000	*p* ^a^	Incidence/person-year	Incidence rate per 1000	*p* ^a^
Total	―	1108/51734	21.4	―	1248/55850	22.3	―
Living arrangement	Living with spouse	871/44890	19.4	*p* < .001	473/31543	15.0	*p* < .001
	Living with non-spousal cohabitants	171/4806	35.6		541/15897	34.0	
	Living alone	66/2039	32.4		234/8410	27.8	
Age	65–69	184/21906	8.4	*p* < .001	148/21341	6.9	*p* < .001
	70–74	309/16272	19.0		244/16573	14.7	
	75–79	322/9216	34.9		357/11301	31.6	
	80–84	199/3384	58.8		307/4955	62.0	
	85 and over	94/955	98.4		192/1680	114.3	
Years of education	<10	656/27872	23.5	*p* < .001	837/34714	24.1	*p* < .001
	≥10	441/23498	18.8		387/20572	18.8	
	Missing	11/364	30.2		24/565	42.5	
Equivalent income	Low	376/17,492	21.5	*p* < .001	407/18,495	22.0	*p* < .001
	Middle	473/23995	19.7		310/18910	16.4	
	High	113/5910	19.1		119/5051	23.6	
	Missing	146/4336	33.7		412/13393	30.8	
Self-rated health	Excellent/good	704/39459	17.8	*p* < .001	749/41359	18.1	*p* < .001
	Fair/poor	387/11844	32.7		469/13540	34.6	
	Missing	17/431	39.4		30/951	31.6	
Presence of illness	No	131/10138	12.9	*p* < .001	120/9535	12.6	*p* < .001
	Yes	941/39942	23.6		1060/43543	24.3	
	Missing	36/1655	21.8		68/2771	24.5	
Geriatric Depression Scale	No depression	619/34677	17.9	*p* < .001	583/32958	17.7	*p* < .001
	Depressive tendency	250/9493	26.3		289/10422	27.7	
	Depression	89/2548	34.9		101/2941	34.3	
	Missing	150/5016	29.9		275/9529	28.9	
Instrumental activities of daily living	Without difficulty	724/39518	18.3	*p* < .001	778/47101	16.5	*p* < .001
	With difficulty	324/10675	30.4		408/6996	58.3	
	Missing	60/1541	38.9		62/1753	35.4	
Subjective cognitive complaints	No	850/44512	19.1	*p* < .001	893/46860	19.1	*p* < .001
	Yes	226/6428	35.2		307/7807	39.3	
	Missing	32/794	40.3		48/1183	40.6	
Body mass index	<18.5	122/2938	41.5	*p* < .001	151/3920	38.5	*p* < .001
	18.5–24.9	767/37038	20.7		732/36996	19.8	
	≥25	177/10653	16.6		240/12722	18.9	
	Missing	42/1105	38.0		125/2212	56.5	
Alcohol consumption	No	512/20451	25.0	*p* < .001	1105/47731	23.2	*p* < .001
	Yes	577/30608	18.9		97/7109	13.6	
	Missing	19/675	28.1		46/1010	45.5	
Smoking habit	None	299/13849	21.6	*p* = .887	1083/50509	21.4	*p* < .001
	Past/current	774/36350	21.3		82/3186	25.7	
	Missing	35/1535	22.8		83/2155	38.5	
Daily walking time	≥30 min	632/32851	19.2	*p* < .001	637/30997	20.6	*p* < .001
	<30 min	411/15574	26.4		451/17498	25.8	
	Missing	65/3309	19.6		160/7355	21.8	
Emotional support received	Yes	851/43165	19.7	*p* < .001	1085/50517	21.5	*p* < .001
	No	183/6589	27.8		92/3151	29.2	
	Missing	74/1980	37.4		71/2182	32.5	
Emotional support provided	Yes	781/41271	18.9	*p* < .001	916/46023	19.9	*p* < .001
	No	240/8182	29.3		236/6740	35.0	
	Missing	87/2280	38.2		96/3086	31.1	
Instrumental support received	Yes	997/48425	20.6	*p* < .001	1090/49835	21.9	*p* = .031
	No	51/1812	28.2		100/4077	24.5	
	Missing	60/1498	40.1		58/1938	29.9	
Instrumental support provided	Yes	927/46692	19.9	*p* < .001	972/49306	19.7	*p* < .001
	No	113/3101	36.4		164/3654	44.9	
	Missing	68/1940	35.0		112/2890	38.8	

^**a**^A log-rank test was used to examine differences among categories for each study variable

Table 4 shows the relationship of living without a spouse (with non-spousal cohabitants or without any cohabitants) for men and women. In Model 1, adjusting for age, both non-spouse household groups were significantly related to the onset of BADL disability in men (hazard ratio [HR] = 1.39 for men only with non-spousal cohabitants; HR = 1.42 for men living alone). When controlling for age, socioeconomic variables, and health variables, HR was 1.38 for men with non-spousal cohabitants, and was slightly increased (HR = 1.45) for those living alone (Model 2). In women, both non-spouse household groups showed no significant relationship with BADL disability in Model 1, and living alone showed a significant relationship in Model 2 (HR =1.19). Among such differences in the relationship between living arrangements and the onset of BADL disability by gender, the interaction effect of living only with non-spousal cohabitants by gender was marginally significant (*p* = .080), indicating a tendency toward a stronger association in men. A sub-analysis to examine differences between the two non-spouse household groups showed no significant difference for men or women in any models. For instance, compared to those who lived only with non-spousal cohabitants, the HR of those living alone was 1.12 (95% confidence interval: 0.82–1.51) and 1.06 (95% confidence interval: 0.90–1.25) in men and women, respectively, controlling for age, socioeconomic status, and health variables.

**Table 4:** Risk of living arrangements on the onset of basic activities of daily living disability

	Men	Women
	HR (95% CI)^a^	HR (95% CI)^a^
Model 1^b^		
Living arrangements		
Living with spouse (Reference)	1.00	1.00
Living with non-spousal cohabitants	1.39 (1.18–1.64)	1.09 (0.95–1.26)
Living alone	1.42 (1.11–1.83)	1.14 (0.97–1.35)
Model 2^c^		
Living arrangements		
Living with spouse (Reference)	1.00	1.00
Living with non-spousal cohabitants	1.38 (1.16–1.63)	1.08 (0.94–1.24)
Living alone	1.45 (1.12–1.87)	1.19 (1.01–1.40)
Model 3^d^		
Living arrangements		
Living with spouse (Reference)	1.00	1.00
Living with non-spousal cohabitants	1.37 (1.16–1.62)	1.09 (0.95–1.25)
Living alone	1.45 (1.12–1.87)	1.19 (1.01–1.40)
Model 4^e^		
Living arrangements		
Living with spouse (Reference)	1.00	1.00
Living with non-spousal cohabitants	1.32 (1.11–1.57)	1.07 (0.93–1.23)
Living alone	1.34 (1.02–1.76)	1.17 (0.98–1.38)
Model 5^f^		
Living arrangements		
Living with spouse (Reference)	1.00	1.00
Living with non-spousal cohabitants	1.31 (1.10–1.56)	1.08 (0.94–1.24)
Living alone	1.35 (1.03–1.77)	1.16 (0.98–1.38)

^a^ *HR* hazard ratio, *CI* confidence interval

^b^Model 1: The effect of living arrangements on the outcome variable controlling for age

^c^Model 2: Model 1 + education + household income + health variables (self-rated health, presence of illness, depression, instrumental activities of daily living, and subjective cognitive complaints)

^d^Model 3: Model 2 + heath-related behavior variables (body mass index, alcohol consumption, smoking habits, and daily walking time) were controlled

^e^Model 4: Model 2 + social support variables (emotional support received, emotional support provided, instrumental support received, and instrumental support provided) were controlled

^f^Model 5: All covariates were controlled

The next three models revealed a substantial excess risk reduction for both non-spouse household groups in men in Model 4, which controlled for social support variables within Model 2. The HR decreased from 1.45 (Model 2) to 1.34 (Model 4) in men living alone, indicating 24.4% excess risk reduction. For men living only with non-spousal cohabitants, a 15.8% excess risk reduction was found in Model 4 compared to Model 2. Additionally, to examine the relative importance of received or provided supports, we conducted an analysis entering the two support-received and two support-provided variables separately in Model 4, and found that the HR of men living alone was 1.40 in the model using only support-received variables, and 1.33 when controlling only for support provided variables. The same tendency was found in men who lived only with non-spousal cohabitants (HR = 1.36 and 1.32, respectively). As for women living alone, Model 4 showed a similar reduction in excess risk of BADL disability of 10.5% compared to Model 2, and social support provision variables almost explained the reduction. The mediation analysis revealed a significant mediating effect of social support in men, which represented 18% and 49% of the total effect in those living with non-spousal cohabitants (*p* = .004) and those living alone (*p* = .036), respectively. On the other hand, mediation effects of health-related variables and those of social support variables in women were non-significant [see Additional file 1: Table S1].

Finally, we conducted a series of sensitivity analyses. First, we limited respondents to those whose IADL were independent at baseline (*n* = 10,892) to avoid reverse causation in which mild disability affected living arrangements. The findings showed that HR for men living with non-spousal cohabitants and those living alone decreased from 1.38 and 1.45 to 1.28 and 1.36, respectively, while HR for women living alone slightly increased to 1.28. Second, we excluded respondents who experienced spousal bereavement within a year (*n* = 537) to avoid the influence of this type of recent stressful life event on BADL disability. The findings showed that HR for men living with non-spousal cohabitants and those living alone were 1.33 and 1.41, respectively, while HR in women living alone decreased from 1.19 to 1.12.

## Discussion

Under the premise of a relatively low rate of needs-driven cohabitation, this study showed a relationship between living arrangements and BADL disability onset, taking into consideration gender differences and cohabitation status of those living without a spouse. Indeed, in our data, only 429 among 13,468 cases (3.2%) moved out during the 9.4 years of the follow-up period. The findings of this study showed that men living without a spouse, regardless of the presence of cohabitants, were more likely to develop BADL disability than those who lived with a spouse, while for women, a significant relationship was found only when they lived alone. Although the interaction effect of living only with non-spousal cohabitants by gender was only marginally significant, this study suggests higher risk in older men compared to women when they live only with non-spousal cohabitants. Several studies have also shown higher risk for BADL disability in older adults living only with non-spousal cohabitants compared to those living with a spouse [12, 15]. However, our study provided new findings that excess risk of living with non-spousal cohabitants in older adults could depend on gender. Our findings suggest that men rely on spousal relationships for protecting their functional health, and that non-spousal cohabitants such as adult children do not compensate sufficiently for the role of the spouse. On the other hand, women may gain health protection from cohabitants, regardless of spousal relationships. Thus, the findings of this study confirmed the need to examine gender-specific risk assessments in the association between social relationships and functional health.

Contrary to previous studies [11, 13, 16, 17] showing a health advantage in older adults living alone compared to those living only with non-spousal cohabitants, the sub-group analysis in this study showed no significant differences in men and women despite using a larger or comparable sample size. This discrepancy might reflect a lower level of reverse causation in this study compared to previous studies, in which decreasing functional ability among older adults living alone leads to cohabitation with non-spousal family members such as adult children.

Our findings also showed a potential pathway between living arrangements and BADL disability. A series of hierarchical analyses (Table 4) showed that social support exchange variables explained more excess risk than did health-related behaviors in men living alone and those living only with non-spousal cohabitants, with reduction rates of 24.4% and 15.8%, respectively. In addition, a subsequent analysis showed the relative importance of support provision rather than support receipt by older adults in decreasing excess risk of BADL disability, even when controlling for predictors such as health. Social support provision is known to improve health in older adults [40, 41]. Older men provide support mostly to their wife, while women provide support to more extended network members such as a spouse, children, or others [22]. Therefore, our findings suggest that living without a spouse could affect disability onset, partially due to lack of opportunities for support provision particularly in men. However, further research is necessary to examine the effect of social support provision and its pathways to functional disability, as the studies in this area are few [42].

Although the lack of social support exchange could explain part of the excess risk for BADL disability, men without spouses still had a significantly higher BADL disability risk than those living with a spouse after controlling for all covariates including mediator variables. This implies that the excess risk of BADL disability in men could be explained by unmeasured factors such as a decrease in social roles or self-efficacy [43].

### Limitations

There were several limitations to this study. First, the onset of BADL was assessed based on the LTCI system; therefore, older adults with functional difficulty who had not applied for the LTCI benefit could have been misclassified as having no functional disabilities. However, such misclassification is less likely for the more severe levels of disability examined in this study [44]. Furthermore, BADL disability in this study was assessed using uniform nationwide criteria based on both a home-visit interview by a trained healthcare professional and a primary physician’s opinion, suggesting that the outcome was less likely to suffer from self-report bias. However, further studies should replicate the findings of this study, using cut-off points reflecting more severe disability for LTCI care-needs or other BADL assessments.

Second, we measured living arrangements as well as covariates (for instance, IADL) only at baseline, which could have changed during the follow-up period of almost 10 years. Although the residential mobility rate in the data we used was very low during the follow-up period, implying less residential moves for support needs, it is still possible that respondents who had lived alone and then started to live with others during the follow-up period may have confounded the findings. In addition, more respondents could have experienced widowhood in the follow-up period, which may have led to an underestimation of the difference between those living with and without spouses in this study. Although we confirmed the excess risk of men living with non-spousal cohabitants and men living alone, even excluding those having a recent spousal bereavement from the analysis, we should further consider the effect of change in living arrangements in older adults to better understand the relationships between living arrangements and health.

Third, generalizability of this study is limited due to a moderate response rate (52.1%); however, the respondents were selected randomly or completely enumerated from 10 municipalities in Japan.

### Implications

Despite these limitations, the findings of our study could provide effective suggestions for the prevention of functional disability in community-dwelling older adults. Focusing on gender differences is important with respect to the role of living arrangements in the prevention of functional disability. Furthermore, more focus should be placed on support needs of older men living without spouses but with non-spousal cohabitants, in addition to those living alone, since older people with any cohabitants tend to be overlooked within the formal support system despite being at risk of BADL decline.

Policy makers and professionals should enhance opportunities for support exchange, particularly support provision by older adults. Social participation is known to be effective for disability prevention [27], and provides opportunities for social support exchange among participants [43]. Therefore, encouraging social participation for older adults at risk for BADL disability due to lack of social support exchange could be helpful. However, since Japanese older men are less likely to participate in these community groups than women [32], it is important to explore the needs and preferences of older men. For instance, a group for walking [32] or manufacturing products [45] might be relatively more acceptable to older men.

Future studies should consider the effect of change in marital status or cohabitation in the analytical model, since studies have shown that these changes could have a negative impact on health [46]. Furthermore, more detailed attention needs to be paid to the role of social support exchanges with spouses in preventing functional disabilities. This may provide practical suggestions regarding possible preventive services for the increasing population of individuals without spouses in aging societies.

## Conclusions

This study examined the relationship between living arrangements and BADL disability onset in community-dwelling older adults, taking into consideration gender differences and cohabitation status of those living without a spouse. The findings showed that older men without spouses were more likely to develop disability onset regardless of cohabitants, while a marginal difference was found only for women living alone, confirming the necessity for gender-specific risk assessments of the effect of social relationships on functional health. Our study also revealed a potential pathway of social support exchange between living arrangements and BADL disability onset, and particularly the role of support giving by older adults. Policy makers and professionals should enhance opportunities for support exchange, and in particular, support giving by older adults who are at risk of disability.

## Additional file

Additional file 1: Table S1. Mediation effects of health-related behavior variables (Model 3) and social support variables (Model 4). (XLSX 11 kb)

## Abbreviations

AGES: Aichi Gerontological Evaluation Study
BADL: Basic activities of daily living
HR: Hazard ratio
JAGES: Japan Gerontological Evaluation Study
LTCI: Long-Term Care Insurance system
